# Effect of S-Allyl –L-Cysteine on MCF-7 Cell Line 3-Mercaptopyruvate Sulfurtransferase/Sulfane Sulfur System, Viability and Apoptosis

**DOI:** 10.3390/ijms21031090

**Published:** 2020-02-06

**Authors:** Patrycja Bronowicka-Adamska, Anna Bentke, Małgorzata Lasota, Maria Wróbel

**Affiliations:** 1 Chair of Medical Biochemistry, Faculty of Medicine, Jagiellonian University Medical College, Kopernika 7, 31-034 Kraków, Poland; patrycja.bronowicka-adamska@uj.edu.pl (P.B.-A.); anna.bentke@uj.edu.pl (A.B.); 2 Institute of Pediatrics, Department of Transplantation, Faculty of Medicine, Jagiellonian University Medical College, Wielicka 265, 30-663 Kraków, Poland; malgorzata.lasota@uj.edu.pl

**Keywords:** S-Allyl-L-cysteine, apoptosis, breast cancer, cystathionine-beta-synthase, gamma-cystathionase, 3-mercaptopyruvate sulfurtransferase, sulfane sulfur

## Abstract

The S-Allyl-L-cysteine (SAC) component of aged garlic extract (AGE) is proven to have anticancer, antihepatotoxic, neuroprotective and neurotrophic properties. γ-Cystathionase (CTH), cystathionine β-synthase (CBS) and 3-mercaptopyruvate sulfurtransferase (MPST) are involved in H_2_S/sulfane sulfur endogenous formation from L-cysteine. The aim of the study was to determine the effect of SAC on MCF-7 cells survival and apoptosis, which is a widely known approach to reduce the number of cancer cells. An additional goal of this paper was to investigate the effect of SAC on the activity and expression of enzymes involved in H_2_S production. The experiments were carried out in the human breast adenocarcinoma cell line MCF-7. Changes in the cell viability were determined by MTT assay. Cell survival was determined by flow cytometry (FC). Changes in enzymes expression were analyzed using Western blot. After 24 h and 48 h incubation with 2245 µM SAC, induction of late apoptosis was observed. A decrease in cell viability was observed with increasing SAC concentration and incubation time. SAC had no significant cytotoxic effect on the MCF-7 cells upon all analyzed concentrations. CTH, MPST and CBS expression were confirmed in non-treated MCF-7 cells. Significant decrease in MPST activity at 2245 µM SAC after 24 h and 48 h incubation vs. 1000 µM SAC was associated with decrease in sulfane sulfur levels. The presented results show promising SAC effects regarding the deterioration of the MCF-7 cells’ condition in reducing their viability through the downregulation of MPST expression and sulfate sulfur level reduction.

## 1. Introduction

Natural sulfur precursor compounds are said to show an anti-cancer potential regarding their ability to inhibit the proliferation, as well as antioxidant possibilities [1]. Much research is conducted with the usage of hydrogen sulfide donors in therapeutic purposes. Among others, breast cancer is researched as one of the most commonly diagnosed malignancies among women [2]. Examples of such a sulfur compound is a naturally occurring sulfonium compound: S-adenosyl-L-methionine (SAM). In recent years, it has been shown to possess antiproliferative properties in various cancer cells. It has also inhibited the proliferation of breast cancer cells MCF-7 by inducing both autophagy and apoptosis [3]. Luo et al. [4] indicated that SAM treatment inhibited cell growth in gastric and colon cancer cells, and the inhibitory effect was significantly higher than that in normal cells. 

SAM reduced the proliferation and induced apoptosis in human head and neck cancer cell lines Cal-33 and laryngeal squamous carcinoma cells (JHU-SCC-011) [5].

**Scheme 1:**
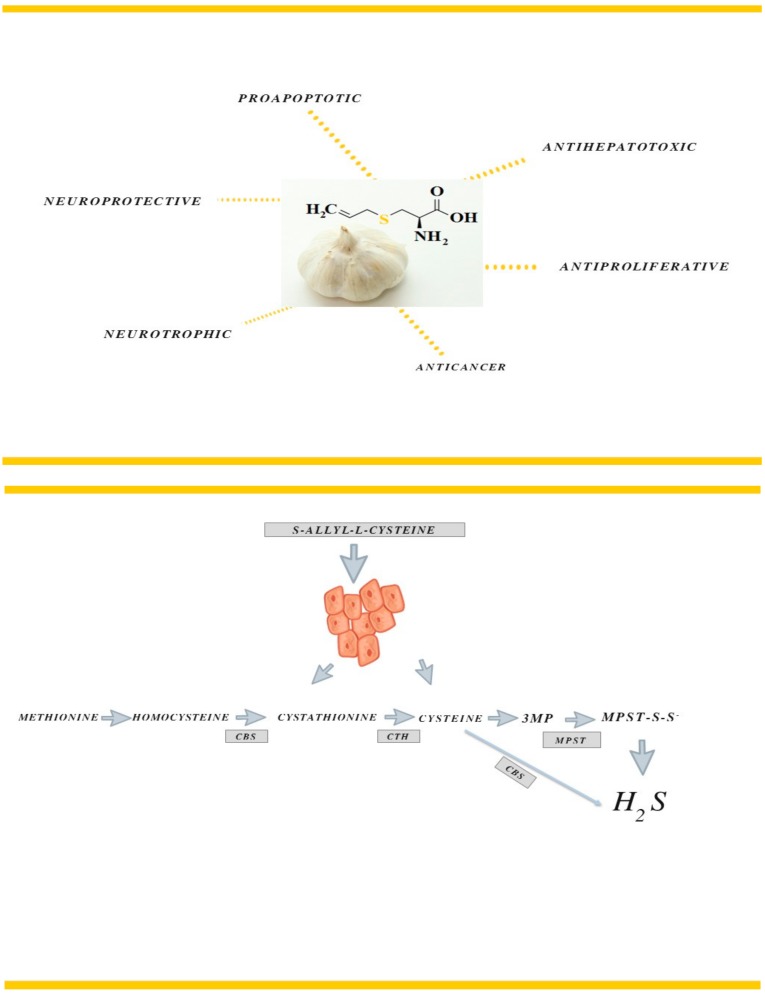
Effect of S-allyl–L-cysteine on the MCF-7 cell line.

Aged garlic extract (AGE) is an odorless garlic preparation containing S-allyl–L-cysteine (SAC) (Scheme 1) as its most abundant compound. A large number of studies have demonstrated the antioxidant activity of AGE and SAC in both in vivo, that is, in diverse experimental animal models associated to oxidative stress, and in vitro conditions, using several methods to scavenge reactive oxygen species (ROS) or to induce oxidative damage [1]. The various data presented its anticancer, antihepatotoxic, as well as neuroprotective and neurotrophic activity [6,7,8]. Welch et al. [9] reported that SAC suppressed the proliferation of LA-N-5 human neuroblastoma cells. In prostate, ovarian, nasopharyngeal and esophageal cancer cell lines, the suppression of invasive growth was proven by Chu et al. [10]. SAC also inhibited the tumor progression in a mouse xenograft model of oral cancer in the experiments presented by Pai et al. [11]. In the hepatocellular carcinoma MHCC97 cell model the authors investigated the SAC influence on the cancer proliferation and metastasis. They reported the induction of apoptosis within the investigated range of concentrations as well as the decrease in the growth and proliferation [12]. This garlic derivative was also shown to suppress proliferation and induce apoptosis in human ovarian cancer cells in vitro [13]. The anticancer effect of S-allyl-L-cysteine via the induction of apoptosis is also mentioned in the Ho research regarding human bladder cancer cells [7]. 

The ongoing search for the presence and role of enzymes involved in sulfur metabolism within the cancer cells has continued over the recent years. The hydrogen sulfide is produced endogenously from L-cysteine in enzymatic pathways catalyzed by: γ-cystathionase (CTH), cystathionine β-lyase (CBS) and by 3-mercaptopyruvate sulfurtransferase (MPST) [14,15]. As described by Sen et al. [16], the cystathionine levels are selectively enriched in human cancer breast cells in comparison to normal cells. The accumulation of cystathionine in breast cancer tissue is said to be associated with the overexpression of CBS. At the same time there is no detection of CTH shown in their research. Both CBS and CTH were not detected in normal breast tissues in the mentioned experiment [17]. The anticancer properties of methionine γ-lyase (MGL) was investigated by Pokrovsky et al. [18]. They described that MGL was active against cancer cells in vitro and in vivo for *Clostridium sporogenes*. Moreover, they showed that doxorubicin and MGL were more effective in A549 human lung-cancer growth inhibition than either agent alone.

The study was conducted to investigate the presence of three enzymes involved in hydrogen sulfide metabolism: CTH, CBS and MPST. We chose the human breast cancer cell line MCF-7 as our in vitro research model. The expression of enzymes (CTH, CBS, MPST) after 24 h and 48 h incubation with selected concentrations of the test compound, SAC, was examined. The changes in the proliferation and apoptosis of cells were also investigated upon the exposure of 800 µM, 1000 µM and 2245 µM SAC. The ability of cells of the MCF-7 line to proliferate in the presence of SAC was checked by MTT assay. The cell viability and induction of apoptosis were examined by flow cytometry (FC) using annexin V and propidium iodide (PI). The changes in the expression of enzymes associated with the hydrogen sulfide metabolism were analyzed using Western blot (WB) with immunohistochemical and chemiluminescent protein detection.

This Scheme 1. was created in the application www.canva.com; 3MP: 3-mercaptopyruvate; MPST: 3-mercaptopyruvate sulfurtransferase; CBS: cystathionine β-lyase; CTH: γ-cystathionase.

## 2. Results

**Figure 1:**
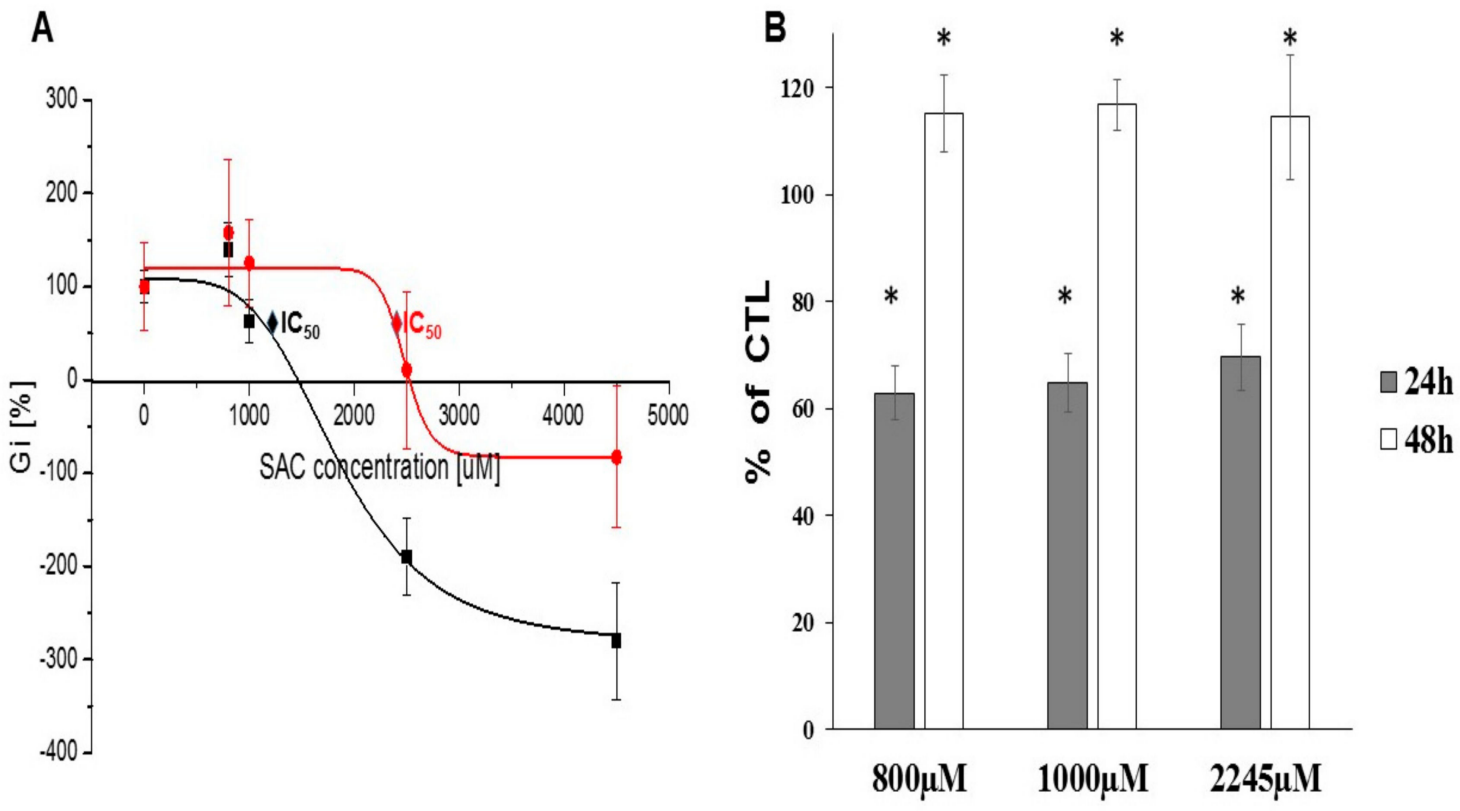
The cell viability after the 24 h and 48 h incubations with 800 µM, 1000 µM and 2245 µM SAC in the MCF-7 cell culture (**A**) generated by OriginPro 9.1 program, (**B**) the MTT assay; Gi: growth inhibition, IC_50_: half maximal inhibitory concentration. The sigmoidal model of effect dependence on SAC dose was adjusted to the obtained data according to the method described in Lasota et al. [19]. All the data of the mean value represent the average of four to five determinations in three experimental groups. Statistical analysis was performed using the Mann–Whitney test (* *p* < 0.05), * *p* < 0.05 vs. the control.

The experiments were carried out on an in vitro model in the human breast adenocarcinoma cell line (MCF-7). The cytotoxic effect upon 24 and 48 h incubation with 800 µM, 1000 µM, and for the highest used concentration, 2245 µM, the SAC was lower than 0.2 %, as measured by LDH Cytotoxicity Detection Kit (Table 1). In the MCF-7 culture, the decrease in cell viability was observed upon increasing SAC concentration and incubation time. The MCF-7 cell line was exposed to SAC for 24 and 48 h, added at the concentrations of 800 µM, 1000 µM and 2245 µM. Three repetitions were performed for each method. The effect of the investigated compounds determined by MTT assay is shown in Figure 1B. The exposure of MCF-7 cells to SAC resulted in a significant dose-dependent suppression of viability compared to the control cultures. Autocrine growth of the investigated cells, determined by MTT assay, was almost completely inhibited at the SAC concentration ≥ 2500 μM after 24 and 48 h. A 50% inhibition of the growth of MCF-7 cells was determined by fitting a sigmoidal model of the dose-dependent effect of the investigated compound. IC_50_ coefficients calculated from the growth inhibition curves (Figure 1A) after 24 and 48 h of incubation were 1185.45 ± 10.93 µM for 24 h and 2382.29 ± 43.69 µM for 48 h, respectively. A significant decrease (*p* < 0.05 for 800 µM, 1000 µM and 2245 µM SAC vs. control) in the cell viability around 40% in relation to the control was observed at 800 µM, 1000 µM and 2245 µM SAC concentrations after 24 h incubation, as measured by MTT assay (Figure 1B). 

**Figure 2:**
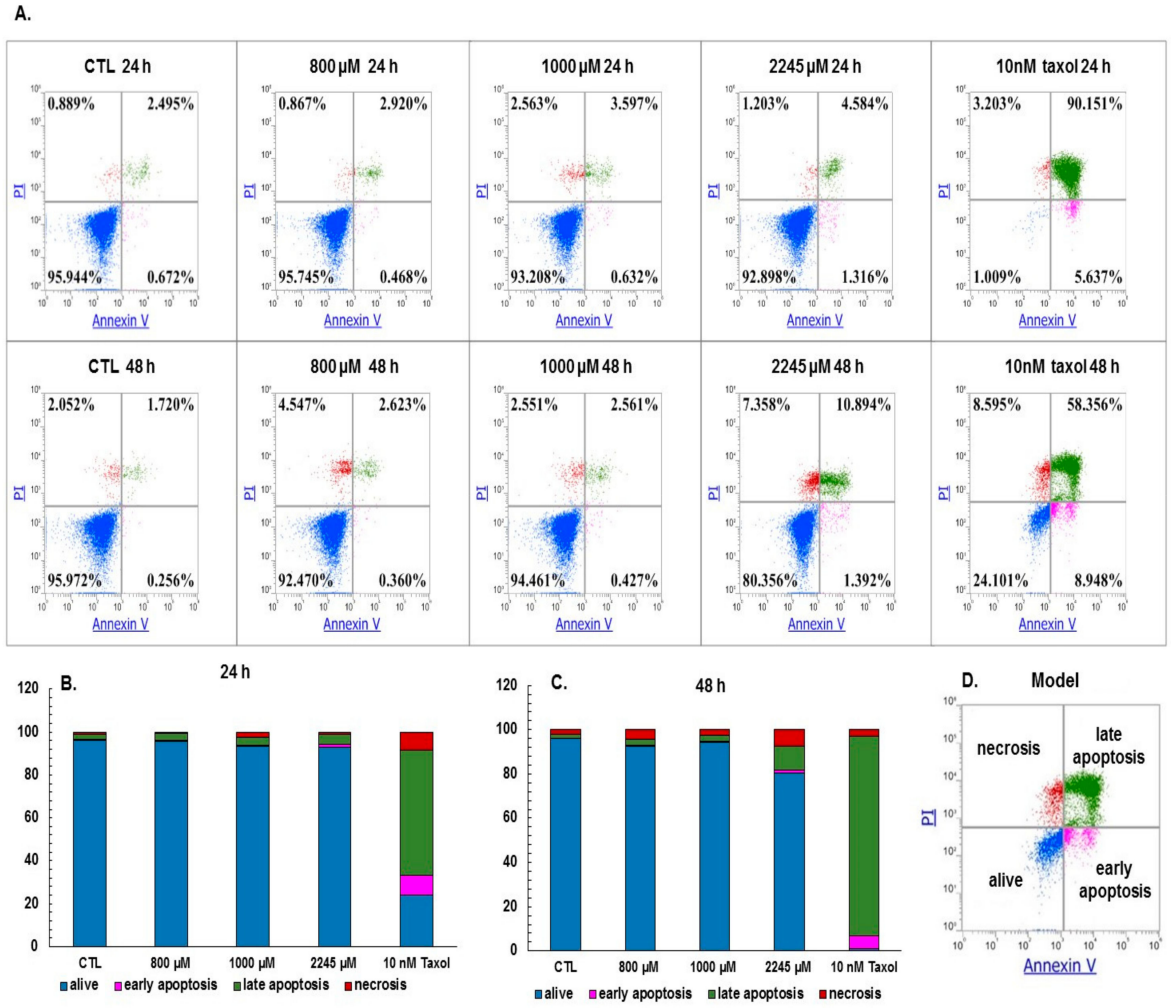
Dot plot showing the result of the annexin/propidium iodide test (**A**) The abscissa axis represents FC fluorescence associated with annexin V, the ordinate axis shows the propidium iodide fluorescence. The effect of SAC in concentrations 800 µM, 1000 µM and 2245 µM and 10 nM taxol (T) on the survival of the individual MCF-7 cell line after the 24 h and 48 h incubations (**B**,**C**), Model: the graphs show the cells: the lower left square shows that which is alive; the upper left square indicates necrosis; the right lower square portrays early apoptosis; the upper right square gives late apoptosis (**D**).

After 24 h and 48 h incubations with the highest concentration of SAC, induction of late apoptosis was observed in the MCF-7 cell line, at the level of 5% and 11%, respectively, measured by the FC method. Moreover, the tested compound had no significant cytotoxic effect on the MCF-7 cells upon all analyzed concentrations. After 24 h incubation with 2245 µM SAC, 93% of viable cells were observed, while 48 h incubation resulted with 80% of viable cells detected (Figure 2A–C). 

**Figure 3:**
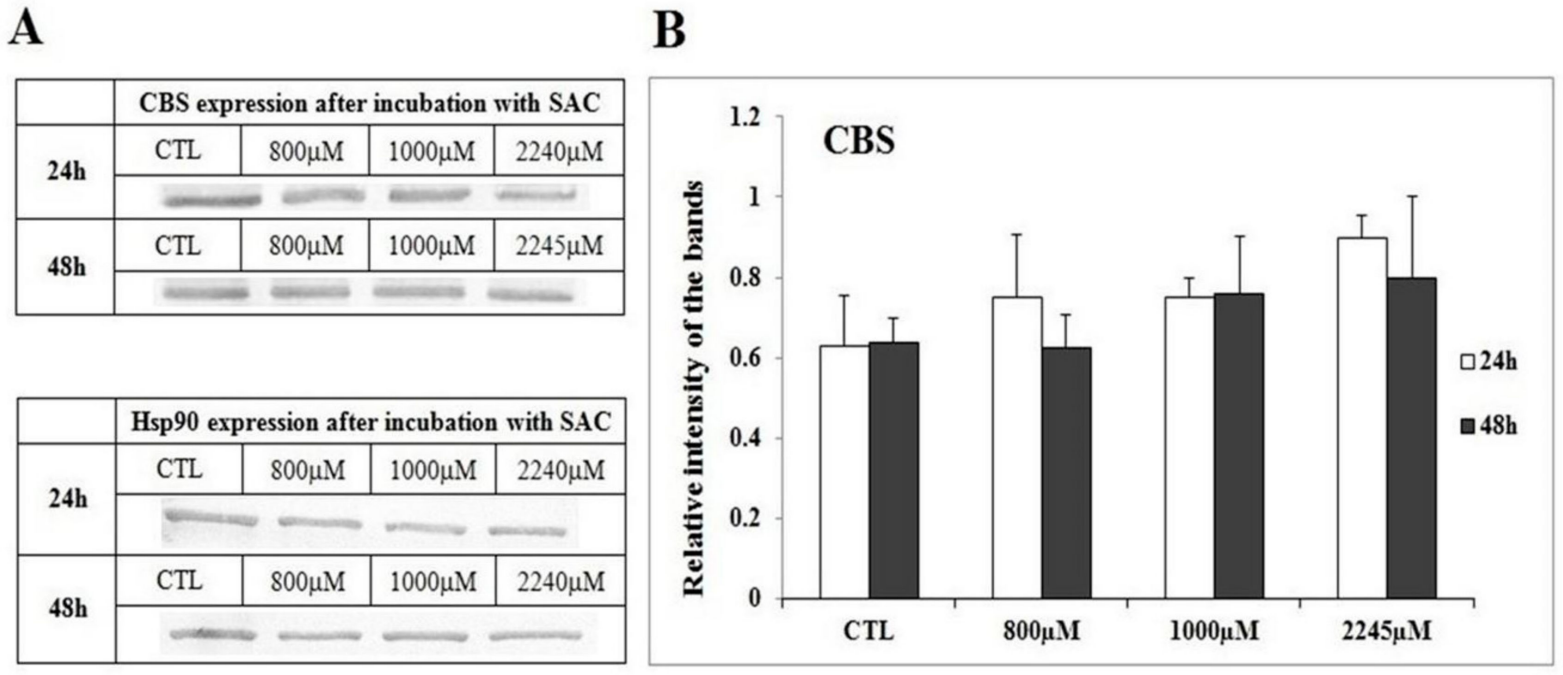
The expression of cystathionine β-synthase (CBS), in the breast cancer cell line (MCF-7) after the 24 h and 48 h incubations with 800 µM, 1000 µM and 2245 µM SAC. The tested concentrations of SAC in culture medium as listed: 800 µM, 1000 µM and 2245 µM, and the control refers to non-treated cells. (**A**) Western blot with immunodetection. Each experiment was done tree times in triplets, and a representative experiment is shown. Hsp90 is used as a control of equal loading. (**B**) Densitometric visualization of expression of CBS. The error bars represent standard deviation.

**Figure 4:**
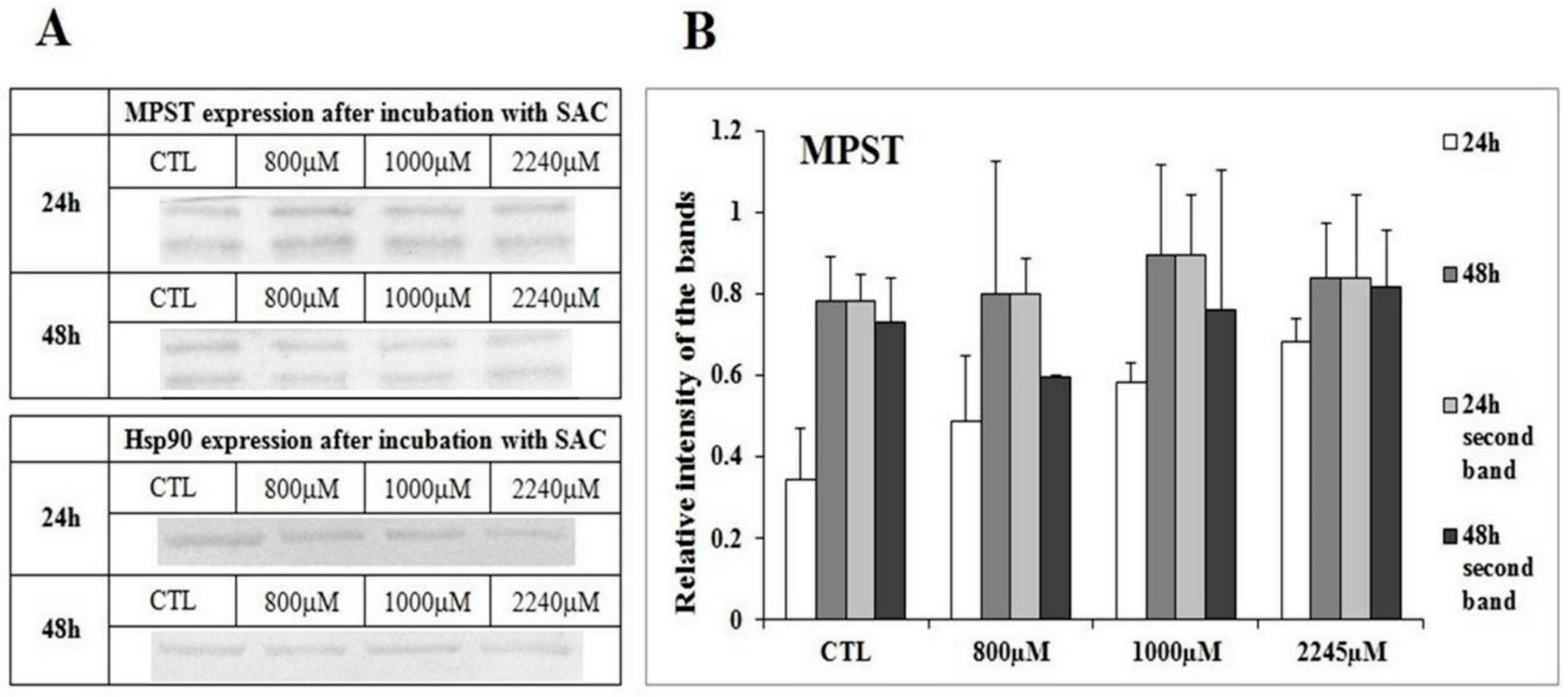
The expression of 3-mercaptopyruvate sulfurtransferase (MPST) in the breast cancer cell line (MCF-7) after the 24 h and 48 h incubations with SAC. The tested concentrations of SAC in culture medium as listed: 800 µM, 1000 µM and 2245 µM, while the control refers to non-treated cells. (**A**) Western blot with immunodetection. Each experiment was done tree times in triplets, and a representative experiment is shown. Hsp90 is used as a control of equal loading. (**B**) Densitometric visualization of expression of MPST. The error bars represent standard deviation.

**Figure 5:**
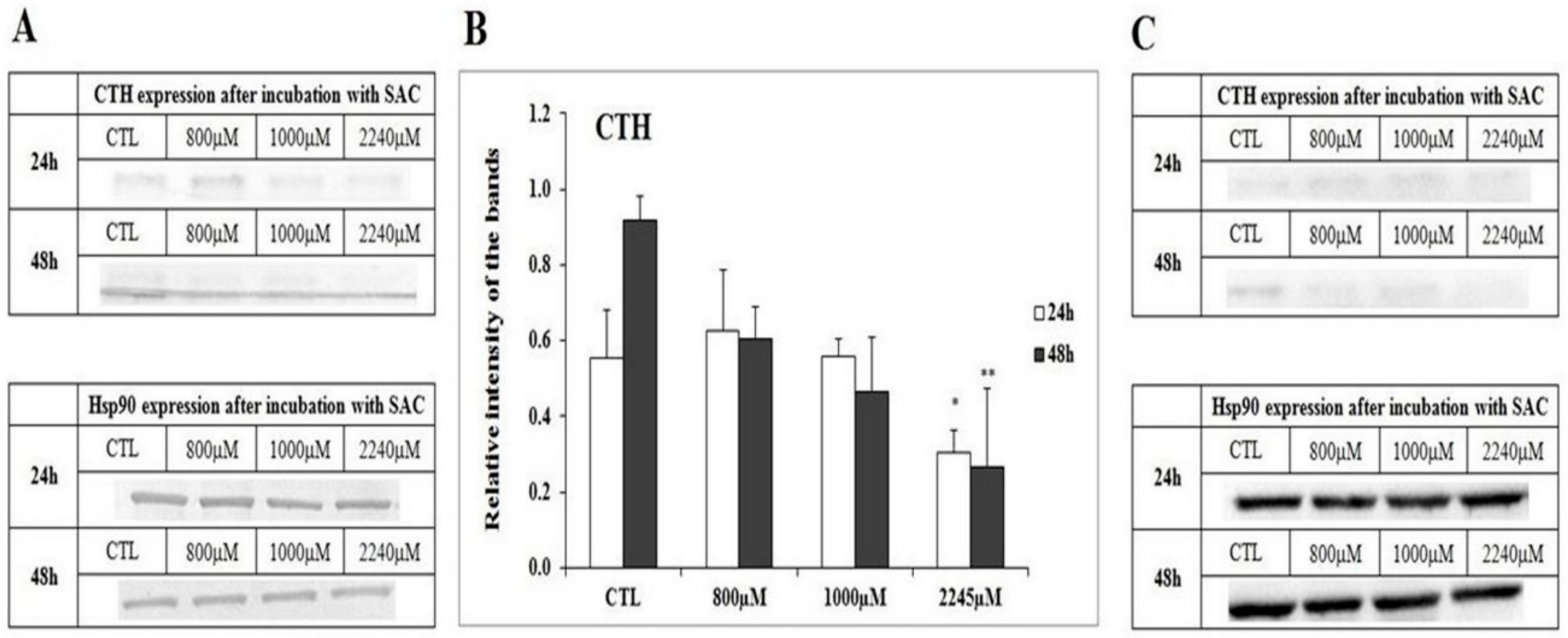
The expression of γ-Cystathionase (CTH) in the breast cancer cell line (MCF-7) after the 24 h and 48 h incubations with SAC. The tested concentrations of SAC in culture medium as listed: 800 µM, 1000 µM and 2245 µM; the control refers to non-treated cells. (**A**) Western blot with immunodetection. Each experiment was done tree times in triplets, and a representative experiment is shown. Hsp90 is used as a control of equal loading. (**B**) Densitometric visualization of the expression of CTH. The error bars represent the standard deviation. (**C**) Western blot with chemiluminescence detection. Statistical analysis was performed using the Mann–Whitney test (* *p* < 0.1 ** *p* < 0.05, 800 µM, 1000 µM, 2245 µM SAC vs. control).

**Figure 6:**
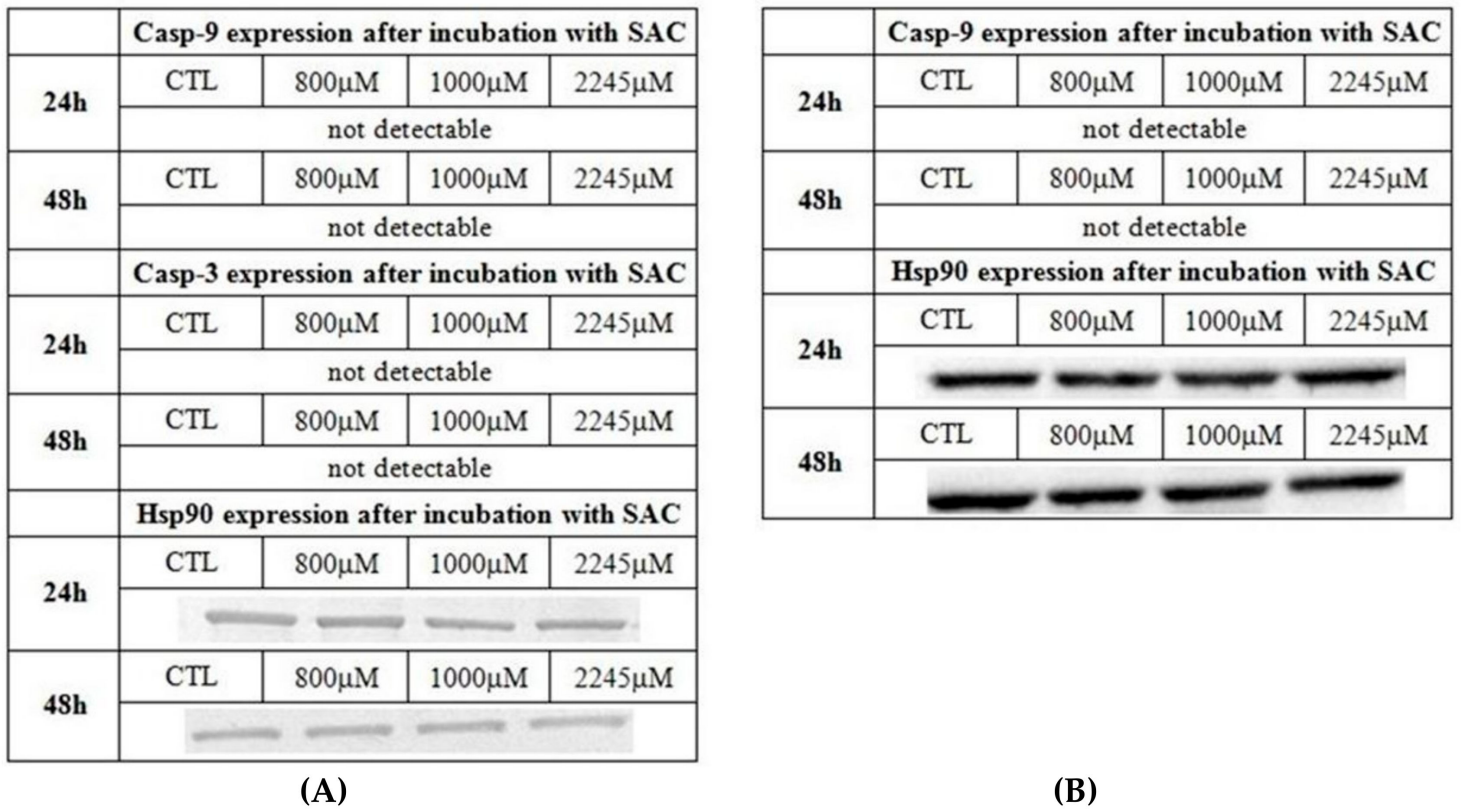
The expression of caspase-3 **(casp-3)** and caspase-9 **(casp-9),** in the breast cancer cell line (MCF-7) after the 24 h and 48 h incubations with SAC. The tested concentrations of SAC in culture medium as listed: 800 µM, 1000 µM and 2245 µM, and the control refers to non-treated cells (**A**) Western blot with immunodetection. Each experiment was done tree times in triplets, and a representative experiment is shown. Hsp90 is used as a control of equal loading. (**B**) Western blot with chemiluminescence detection.

Different concentrations of SAC were added for 24 and 48 h in to the cell culture, and the WB method was chosen to confirm the expression of the analyzed enzymes on the protein level. The immunodetection method was used for band visualization (Part A of Figure 3, Figure 4, Figure 5 and Figure 6). Each experiment was repeated at least three times with a minimum of three WB runs for each experiment. Hsp90 is used as a control of protein loading. The densitometry analysis for all bands was conducted, and the results were presented as a relative intensity on the bands within the each figure (Part B of Figure 3, Figure 4 and Figure 5). 

While investigating the effect of SAC upon expression, we did not observe statistically significant changes of CBS and MPST expressions after the 24 h and 48 h incubations with 800 µM, 1000 µM and 2245 µM SAC (Figure 3 and Figure 4). The changes in the level of the tested enzymes were observed for CTH, especially for the highest concentrations of the SAC used. Thus it was concluded that SAC may reduce the CTH expression. We confirmed a statistically significant decrease in the relative intensity for the CTH bands after the 24 h and 48 h incubations with 2245 µM of SAC with densitometry measurements (Figure 5B). 

Additionally, the early apoptosis mitochondrial pathway caspases: 3 and 9 were included into the experiment. There were no detections of both mentioned caspases as measured by WB with colorimetric detection (Figure 6). To confirm the changes in CTH expression and the lack of caspase-9 detection, the same samples were run again via WB protocol, but a different, more sensitive chemiluminescence method of detection was used. The results from the previous experiment were confirmed, and are shown in Part C of Figure 5 and Part B of Figure 6. 

In Table 2 and Table 3 the activity of CTH, MPST and the level of sulfane sulfur in MCF-7 cells after the 24 and 48 h incubation with 800 µM, 1000 µM and 2245 µM SAC concentrations are presented. The CTH and MPST activity involved in H_2_S production in the non-treated MCF-7 cells (control) was confirmed in the experiment. The specific CTH and MPST activity was expressed in nmol of product produced during 1 min per 10^6^ cells. After the 24 hour incubation, a significant decrease in MPST activity and the sulfane sulfur level at 2245 µM concentrations of SAC were observed. More than twofold lower CTH activity at 800 µM SAC concentration and fivefold lower CTH activity at 1000 µM SAC concentration after the 24 h incubation were estimated in the MCF-7 cell, as compared to the control. On the other hand, after 24 h of incubation with the highest SAC concentration (2245 µM), the CTH activity returned to the control activity, despite the decreasing CTH expression, as measured by WB (Table 2, Figure 5A–C). Slightly different changes were observed after the 48 h incubation with SAC. MPST activity changes were not significant in comparison to the 24 h incubation; only the increase in MPST activity vs. the control with 1000 µM SAC concentration was observed. Threefold lower CTH activity at 1000 µM SAC concentration was confirmed. Similarly, after the 24 h incubation with the highest SAC concentration, the CTH activity increased to the activity observed in the control. A significant decrease in MPST activity at 2245 µM SAC vs. 1000 µM SAC was associated with decreased sulfane sulfur levels. The level of sulfane sulfur increased twofold vs. control at 800 µM and 1000 µM SAC concentrations, and decreased twofold at the highest SAC concentration after 48 h incubation (Table 3). CBS activity was not determined in this experiment, as there were no CBS expression changes detected by WB. CBS activity in non-treated MCF7 cells was established at the level of 1.3 ± 0.01 pmol produced during 1 min per 10^6^ cells. 

## 3. Discussion

Evasion of apoptosis is one of the hallmarks of cancer that allows the tumor continuous growth and progress [20]. Induction of apoptosis via certain substances is a widely known approach to reduce the amount of cancer cells, and is a common way in fighting the cancer progression [21,22]. Efficient induction of apoptosis in lung, breast (MCF-7), cervical and colon cancer cells were shown after the treatment with paclitaxel [23]. The apoptosis upon *E. ferox salisb* exposure was observed in vivo in Balb/c-nu mice bearing A549 xenografts, and results suggested a proapoptotic effect in a p53-dependent manner [24].

Similarly to the above examples, new substances are selected as the potential apoptosis inductors in the breast cancer MCF-7 cell model, and various substances are tested. Induction of apoptosis in human breast cancer cells via the caspase pathway by Vernodalin isolated from *Centratherum anthelminticum* was shown by Looi et al. [25]. The promising effects were shown for substances such as govaniadine isolated from the *Corydalis govaniana* root walls where cytotoxic and pro-apoptotic effects were described in the MCF-7 cell model [26]. Apoptosis induction in this cell model was also described by Oladghaffari et al. [27], as well as Yang et al. [28]. In the Tor et al. [29] studies, apoptosis was effectively induced by putting MCF-7 cells to oxidative stress via the ethyl acetate extract of *D*. *suffruticosa.* In the Pilco-Ferreto and Calaf [30] study, authors indicated that doxorubicin induced apoptosis by upregulating Bax, caspase-8 and caspase-3, and by the downregulation of Bcl-2 protein expression in MCF-10F, MCF-7 and MDA-MB-231 breast cancer cells.

In our studies the efficient induction of late stages of apoptosis was shown upon 2245 µM exposure after the 24 h and 48 h incubations with SAC in culture medium, as measured by the FC method. In the same time, no expression of the caspase-3, which is the early apoptosis stage indicator, was determined as measured by WB. In the experimental model it could be explained with MCF-7 being often described as a caspase-3 deficient cell line. As reported by Shunsuke et al. [31], deficiency of caspase-3 in MCF-7 cells resulted in blocking bax-mediated nuclear fragmentation, but not in cell death in general [31,32]. Despite MCF-7 cells being deficient or absent of caspase-3, they can still undergo apoptosis through an intrinsic apoptotic pathway and release cytochrome c to activate caspase-9 [33,34]. 

As there was no detection of caspase-9 in our experiment (as measured by WB with two different detection methods, as well as the usage of anti-caspase-9 antibody from two different manufacturers), it can be assumed that after such a period of incubation with SAC, the cells are either not undergoing the early stage of apoptosis, and/or they are already in the late stage of this process. It was confirmed as shown by the FC method that the early apoptotic cells are barely present, and the late stage apoptotic cells are detected in the majority upon the exposure with 2245 µM SAC. Thus, findings of this study may contribute to the statement of the potential anticancer role of S-allyl-L-cysteine. We demonstrated that SAC exhibits a potential anti-cancer effect on the MCF-7 breast cancer cell line, as supported by the results regarding the induction of apoptosis The contribution is additionally enriched by showing the proliferation changes in our experimental incubation. Various scientific studies confirmed that SAC may be potentially therapeutic in the strategy to combat breast cancer via lowering the proliferation rate, and was shown to be effective after short and long term treatments [10,35]. As described by Gapter et al. [35] at high doses, SAC showed a strong toxic effect and the ability to suppress breast cancer cell migration and adhesion, as well as to reduce the processes involved in breast cancer metastasis. In further study, SAC was shown to suppress cancer cell growth over non-malignant cells [10]. Tang et al. [36] confirmed that SAC caused a significant decrease in tumor growth. They reported lowering the rate of proliferation, suppressing the PI3K/Akt pathway and changing the NF-kB and cyclooxygenase-2 expression in the nude mice model implanted with non-small cell lung carcinoma A549 cells. 

In this study we considered SAC as a hydrogen sulfide donor. MPST, CBS and CTH are the enzymes responsible for H_2_S production, which can be released from SAC. These enzymes of the trans-sulfuration pathways are also responsible for providing some amount of cysteine, which is an essential substrate for intracellular GSH production that exhibits antioxidant properties and prevents damages caused by reactive oxygen species (ROS) [8]. Scientific reports describe the CBS and CTH roles in breast cancer models [16,37]. The CBS expression measured by different methods was described in breast cancer tissues by Sen et al. [17], but they reported no CTH presence in the collected tissue samples. On the contrary, You et al. [37] described the CTH expression in MCF-7 cells and provided the results supporting its significant role in the breast cancer development. Furthermore, there is no sufficient data regarding MPST functions in the MCF-7 cell line, and the information to determine the MPST role in the tumor process. The potential role of MPST in cancer cells was recently discussed by Augsburger and Szabo [38]. In our results, CBS and CTH presence and activity were shown in the MCF-7 in vitro model, and this correlates with the literature data. Additionally, the experiments confirmed both the expression and activity of MPST in the studied MCF-7 cell line. There is no study in literature on the SAC effect on the mentioned enzymes involved in H_2_S production in the MCF-7 cell line. So the findings of this study are an addition to the knowledge on the changes of these enzymes’ expression upon SAC exposure and on the enzymatic activity profile of this breast cancer cell line. 

Sen et al. [16] have shown that CBS silencing inhibited the breast cancer cell growth in the presence of activated microphages. They suggested that CBS-derived H_2_S might have protected breast cancer cells from the attack of microphage instead of directly promoting cell growth. The pro-cancer effect of CBS in human breast cancer has been further consolidated by a recently reported association between the 844ins68 polymorphism in the CBS gene and the occurrence of breast cancer [39]. Studies presented in our report suggest that CBS expression does not change under the influence of SAC. Meanwhile, we confirmed that SAC reduced CTH expression, but without changes in the sulfane sulfur level. In the already mentioned paper by You et al. [37], these authors reported CTH expression upregulation in both in vivo and in vitro breast cancer models, and combined it with promotion of the proliferation and migration of breast cancer cells. CTH function was said to depend on the STAT3 signaling pathway. The pathway is a regulator of key cell functions, including cell growth in the variety of human cancer cells [37]. Given above, and the fact that CTH expression decreases upon the exposure of the highest SAC concentration, this all may suggest SAC’s beneficial involvement in the breast cancer MCF-7 cell line. 

High MPST activity and expression have been proven in numerous cancer cell lines and tissues, including human prostate tissue, urothelial carcinoma cells of bladder, colon cancer and human glioblastoma-astrocytoma and neuroblastoma cell lines [15,40,41,42]. As shown in our paper, the MCF-7 line is characterized by relatively high MPST levels. Also, SAC used as a donor of H_2_S caused a decrease in MPST activity. This decrease in MPST activity was associated with lower sulfur levels. Sulfane sulfur species [43] can be produced from L-cysteine. Cysteine aminotransferase and MPST consecutively metabolize L-cysteine to produce sulfane sulfur-containing compounds such as: thiosulfate, organic polysulfides and persulfide. Sulfane sulfur atoms are also described as products of H_2_S oxidation, and as those recognized as mediating some of the H_2_S biological effects. Reactive sulfane sulfur is likely involved in protein sulfhydration and protecting cells from oxidative stress [38]. Augsburger and Szabo [38] announced that the MPST/H_2_S system is involved in supporting cancer cell proliferation, as well as suggested a potential regulatory role in bioenergetics and cell-signaling functions. Therefore, the exact role of MPST in cancer development remains still not elucidated, and could be a worthy investigating area and topic to open the discussion for the future [44]. Sulfane sulfur plays a critical role in redox homeostasis, and as described by Shinkai et al. [44], endogenous sulfane sulfur could be used as a chemo-preventive agent against electrophilic stress. It was mentioned that the knockdown of sulfane sulfur-producing enzymes resulted in the increased electrophile-mediated toxicity [44]. 

## 4. Materials and Methods

### 4.1. Chemicals

Pyridoxal phosphate (PLP), β-Nicotinamide adenine dinucleotide reduced disodium salt hydrate (NADH), L-Lactic dehydrogenase (LDH), 3-mercaptopyruvate acid sodium salt, D,L-dithiothreitol, (DTT), N-ethylmaleimide (NEM), sodium dihydrogen phosphate dihydrate pure, sodium sulfite, sodium chloride, Folin-Ciocalteu’s phenol reagent, iron (III) nitrate nonahydrate, sodium thiosulfate pentahydrate, sodium carbonate, insulin, Coomassie Blue G250,Tris, glycine, sodium dodecyl sulfate, ammonium persulfate and acrylamide were obtained from Sigma-Aldrich (St. Louis, MO, USA), while 2-mercaptoethanol was purchased from Fluka Chemie GmbH (Buchs, Switzerland). Ethanol and 38% formaldehyde, 65% nitric acid, 38% hydrochloric acid, ammonia solution 25% pure, sodium potassium tartrate, copper sulfate pentahydrate, potassium dihydrogen phosphate, ferric chloride and sodium hydroxide were from Polskie Odczynniki Chemiczne S.A. (Gliwice, Poland), DMEM/High glucose, trypsin 0.25%, fetal bovine serum and penicillin-streptomycin solution were purchased from Thermo Fisher Scientific (Waltham, MA, USA). Potassium cyanide was from Merck Sp. z o.o. (Warszawa, Poland). RIPA buffer was from TermoScientific (Rockford, USA). Antibodies: anti-CBS and anti-CTH were from Abnova (Taiwan), anti-MPST was from GeneTex (Taiwan), β-actin from Sigma-Aldrich (Poznan, Poland), Anti–Hsp90 from Cell Signaling Technology (Danvers, MA, USA), caspase-3 and caspase-9 from Proteintech and Cell Signaling Technology (Danvers, MA; Chicago, IL, USA), Alkaline Phosphatase-conjugated goat anti-rabbit IgG antibody and anti-mouse IgG antibody were from Proteintech (Chicago, IL, USA); HRP-conjugated goat anti-rabbit IgG antibody and anti-mouse IgG antibody were from Cell Signaling Technology (Danvers, MA; Chicago, IL, USA). NBT/BCIP (Nitroblue tetrazolium chloride/5-Bromo-4-chloro-3-indolyl phosphate, toluidine was from Roche (Warszawa, Poland), SignalFire™ Elite ECL Reagent from Cell Signaling Technology (Danvers, MA; Chicago, IL, USA).

### 4.2. Cell Culture

The human breast adenocarcinoma cell line MCF-7 was purchased from the American Type Culture Collections (ATCCs). Cell lines were grown in a monolayer in Dulbecco’s Modified Eagle Medium (DMEM) supplemented with 10% fetal bovine serum, antibiotics (100 U/mL penicillin and 100 µg/mL streptomycin) and 0.01 mg/mL human recombinant insulin in plastic culture dishes (100 mm in diameter), at 37 °C in a humidified atmosphere containing 5% CO_2_. 

### 4.3. Cell Treatment

Cells were seeded on 96-well plates (1.2–1.8 × 10^4^ cells per well) or 6-well plates (4 × 10^5^ cells per well). 24 h after seeding, growth medium was replaced with a medium containing the appropriate SAC concentration. The final applied concentrations of each treatment were 800 µM, 1000 µM and 2245 µM for 24 and 48 h. Untreated cells in culture medium were used as a negative, non-treated control.

### 4.4. Cytotoxicity of Reagents

The cells were seeded in triplicates into 96-microwell plates at density of 16–18 × 10^3^ cells/well and incubated for 24 h and 48 h with or without 800 µM, 1000 µM and 2245 µM SAC in DMEM medium supplemented with 10% FBS and insulin. LDH Cytotoxicity Assay Kits (Thermo Fisher Scientific, Waltham, MA, USA) provide a reliable colorimetric assay that can be used to quantitatively measure LDH released into the media from damaged cells as a biomarker for cellular cytotoxicity according to the manufacturer’s protocol. 

### 4.5. Cell Viability by MTT Assay

For the determination of cell viability by MTT (3-(4,5-dimethylthiazol-2-yl)-2,5-diphenyltetrazolium bromide) assay, the cells were seeded on 96-well plates at a concentration of 20 × 10^3^ cells/well (MCF-7 cells) in 200 µl DMEM supplemented as reported above. After the 24 h or 48 h incubations with SAC, 10 µL MTT (5 mg/1 mL) was added, and then the incubation lasted for 3 h. Subsequently, lysis buffer (10%SDS/0.01 M HCl) was added to the cells. Cells were incubated all night. This colorimetric method uses the reduction of MTT to measure cellular metabolic activity as a proxy for cell viability. Viable cells contain NAD(P)H-dependent oxidoreductase enzymes which reduce the MTT reagent to formazan, an insoluble crystalline product with a deep purple color. The darker the solution, the greater the number of viable, metabolically active cells. The absorbance was measured at 570 nm using an Epoch Microplate Spectrophotometer (BioTek Instruments Inc, Winooski, VT, USA).

### 4.6. Flow Cytometry (FC) 

The cells were grown to 90% confluence, treated with the right SAC concentration and incubated for 24 and 48 h. The tested concentrations of SAC in culture medium were as listed: 800 µM, 1000 µM and 2245 µM. After the incubation, cells were harvested and washed twice with PBS. Cells were counted in a Bürker chamber and suspended: 1 × 10^6^ cells/mL in a binding buffer (Becton Dickinson, Franklin Lakes, NJ, USA). The analysis was carried out according to the FITC Apoptosis Detection Kit protocol BD Pharmingen™ (Becton Dickinson, Franklin Lakes, NJ, USA). 100μL of cell suspension (1 × 10^5^ cells/mL) was transferred to the cytometric tubes, 5 μL of Annexin V was added, and samples were left in the dark for a 15-min incubation at room temperature. After the incubation, 5μL of propidium iodide and 400 μL of buffer solution were added prior to the cytometric analysis alone. The results were analyzed on Attune Nxt Flow cytometer (Thermo Fisher Scientific, Waltham, MA, USA).

### 4.7. Western Blot Analysis

The cells were suspended in RIPA buffer, containing proteinase inhibitor cocktail, sonicated 3 × 5 s at 4 °C (Bandelin Sonoplus GM 70, BANDELIN electronic GmbH & Co. KG, Berlin, Germany) and centrifuged at 14,000 × g for 15 min, and the supernatants were used for further analysis. The protein load for electrophoresis was 20 µg per well. SDS-PAGE electrophoresis and electro-transfer on the PVDF membrane was conducted according to the Bio-Rad protocol on the Mini-PROTEAN Tetra Cell system. Membranes were washed with TBS-Tween, then blocked with non-fat milk over 1.5 h. After washing with TBS-Tween, all membranes were cut into three or four parts, taking into consideration the protein ladder added on the first and last well. The appropriate piece of membrane was incubated with the proper primary anti-antibody and secondary antibody. The relative amounts of MPST CBS and CTH were determined by Western blotting using the appropriate antibody: anti-CBS (1:1000), anti-CTH (1:1000), anti-MPST (1:1000). The relative amounts of casp-3 and casp-9 were determined using: anti-casp-3 (1:200), anti-casp-9 (1:200; 1:1000). Anti–Hsp90 (1:2000) antibody was used to control equal loading. Hsp90 was chosen due to its abundant presence in many cellular models, including MCF-7. It is described as one of the most abundant proteins in all types normal and cancer cells, to be precise, 1%–2% of the total cellular proteins [45,46]. Hsp90 was also used due to its location on the membrane after separation (molecular weight 90 kDa) that allowed for the analysis of as many as four different proteins from one membrane (molecular weight: MPST 34 kDa, CTH 42–45 kDa and CBS 63 kDa). 

Proteins of interest were detected with (a) the colorimetric method by using Alkaline Phosphatase-conjugated goat anti-rabbit IgG antibody (1:200) and Alkaline Phosphatase-conjugated goat anti-mouse IgG antibody (1:200), or with (b) the chemiluminescent method by using HRP-conjugated goat anti-mouse IgG antibody (1:10000) or HRP-conjugated goat anti-rabbit IgG antibody (1:10000). Proteins were visualized by (a) immunodetection with NBT/BCIP staining solution/substrate or (b) chemiluminescence with SignalFire™ Elite ECL Reagent. Each experiment was repeated a minimum of three times, with a minimum of three WB runs with similar results. The representative experiments are shown.

### 4.8. Densitometric Evaluation of Western Blot bands

The photos saved in the jpeg format (ChemiDoc^TM^ MP Imaging System with Image Lab Software, version 6.0, Bio-Rad, Hercules, California, CA, USA) were used for densitometry analysis. The densitometry data for band intensities in different experiments was generated by analyzing the WB images on the Gene Tools Software version 4.3.7.0 (Syngene, Cambridge, UK). The single bands were referred to the band for load-control (Hsp90) and were normalized within the experiment. The data for all bands, from all experiments, were added to the mean that is represented as the relative intensity on the y axis (in relation to the load-control for the same row). The error bars represent the standard deviation from all measurements within all experiments and technical repetitions.

### 4.9. Enzymes Assay

#### 4.9.1. Cell Homogenization 

MCF-7 cells (3.5–5 × 10^6^ cells) were suspended in 0.1 M phosphate buffer pH 7.5, in the proportion 1 mln cells/0.07 mL of the buffer, sonicated 3 × 5 s at 4 °C (Bandelin Sonoplus GM 70, Bandelin electronic GmbH & Co. KG, Berlin, Germany). After centrifugation at 1600× g for 10 min, the supernatant was used for the determination of protein concentration, sulfane sulfur levels and the activity of MPST and CTH. 

#### 4.9.2. MPST Activity 

MPST activity was assayed according to the method of Valentine and Frankelfeld [47] following the procedure described in our earlier paper [48]. The incubation mixture contained: 250 µl, 0.12 M sodium phosphate buffer, pH 8.0, 50 µl, 0.5 M sodium sulfite, 50 µl 0.15 M D,L-dithiothreitol (DTT), 50 µl supernatant, 50 µl distilled water and 50 µl 0.1 M 3-mercaptopyruvate acid sodium salt in the final volume of 500 µl. The mixture was incubated for 15 min., 250 µl of 1.2 M PCA was added to stop the reaction.

Samples were centrifuged at 1600× g for 5 min, and 100 µl of supernatant was transferred to a 1350 µl mixture that contained: 1200 µl, 0.12 M sodium phosphate buffer, pH 8.0, 100 µl 0.1 M N-ethylmaleimide (NEM), 50 µl NADH 5 mg/mL. After equilibration at 37 °C, 2.5 µl of (7 IU) L-lactate dehydrogenase (LDH) was added, and the decrease in absorbance was measured at 340 nm. MPST activity was expressed as nmoles of pyruvate (PA) produced during 1 min incubation at 37 °C per 1 mg of protein. 

#### 4.9.3. CTH Activity

CTH activity was determined by Matsuo and Greenberg’s method [49], with modifications described by Czubak et al. [50]. The incubation mixture contained: 25 µl 1.3 mM PLP, 25 µl 0,02 mM EDTA, 250 µl 45 mM cystathionine in 0.1 M phosphate buffer, pH 7.5 (2.5 mg cystathionine per sample) and 75 µl supernatant and 0.1 M phosphate buffer, pH 7.5, containing 0.05 mM 2-mercaptoethanol in a final volume of 650 µl. The reaction was stopped after 15 min of incubation at 37 °C by placing 125 µl of incubation mixture in 25 µl 10% PCA. Samples were centrifuged at 1600× g for 10 min, and 25 µl of supernatant was transferred to 625 µl 0.194 mM NADH and kept at 37 °C. Control samples (without 45 mM cystathionine) were prepared in the same way as the examined samples. After 10 s of the measurement (absorbance at 340 nm), 25 µl (9.06 IU) LDH was added and measurement was continued to 180 s. The difference between the initial value of absorbance (before adding of LDH) and the lowest value (after adding of LDH) corresponded to the amount of alpha–ketobutyrate formed in the course of the cystathionase reaction. CTH activity was expressed as nmoles of alpha-ketobutyrate formed during 1 min incubation at 37 °C per 1 mg of protein.

#### 4.9.4. Sulfane Sulfur 

Sulfane sulfur was determined by the method of Wood [51], based on cold cyanolysis and colorimetric detection of the ferric thiocyanate complex ion. The sulfane sulfur level was expressed as nmoles of SCN^-^ produced per 1 mg of protein.

#### 4.9.5. Protein 

Protein concentration was determined by the method of Lowry et al. [52], using crystalline bovine serum albumin as a standard. Protein concentration by Bradford assay was used for the determination of protein in Western blot analysis [53].

### 4.10. Statistical Analysis

Statistical analyses were performed using the Statistica, version 13.3 (TIBCO, Software Inc., Palo Alto, CA, USA). The results were expressed as means ± standard deviation (SD). The significance of the differences between controls and our investigated samples were calculated using the Mann–Whitney test. 

## 5. Conclusions

The results presented in this work show the promising effect of SAC regarding the deterioration of the MCF-7 cells condition and reducing their viability through the downregulation of MPST expression and sulfate sulfur level reduction, so through the MPST/sulfane sulfur system.

## Figures and Tables

**Table 1:** The cytotoxicity effect of S-Allyl-L-cysteine (SAC) in concentrations 800 µM, 1000 µM and 2245 µM in the human breast adenocarcinoma cell line MCF-7 after 24 h and 48 h incubations.

	24 h	48 h
	% of Cytotoxicity	
800 µM	0	0
1000 µM	0	0
2245 µM	0.2	0

**Table 2:** The mean value of MPST, CTH activity and sulfane sulfur level in the MCF-7 cell line after a 24 h incubation with SAC.

MCF-7 Cell Line 24 h	MPST	CTH	Sulfane Sulfur
nmol·10^6^·cells^−1^·min^−1^			nmol·10^6^·cells^−1^
**CTL**	2714 ± 904	25 ± 9	826 ± 249
**800 µM**	3042 ± 310	11 ± 6*	949 ± 279
**1000 µM**	3636 ± 818	5 ± 1*	999 ± 198
**2245 µM**	1828 ± 389 *^Δ^	25 ± 16	497 ± 100 *^Δ^

Values determined in MCF-7 cells homogenates were combined to calculate the mean value. All the data of the mean value represent the average of four to five determinations in three independent experiments. Values in Table 2 were summarized as a mean ± standard deviation of the mean. Statistical analysis was performed using a Mann–Whitney test (* *p* < 0.05). * *p* < 0.05 800 µM, 1000 µM, 2245 µM SAC vs. control, ^Δ^ *p* < 0.05, 2245 µM SAC vs. 1000 µM SAC.

**Table 3:** The mean value of MPST, CTH activity and sulfane sulfur level in the MCF-7 cell line after a 48 h incubation with SAC.

MCF-7 Cells 48 h	MPST	CTH	Sulfane Sulfur
nmol·10^6^·cells^−1^·min^−1^			nmol·10^6^·cells^−1^
**CTL**	3261 ± 798	30 ± 10	929 ± 194
**800 µM**	2580 ± 761	50 ± 10*	1731 ± 426*
**1000 µM**	4229 ± 830*	10 ± 5*	1905 ± 430*
**2245 µM**	2019 ± 382 ^Δ^	20 ± 1	571 ± 116*^Δ^

Values determined in MCF-7 cells homogenates were combined to calculate the mean value. All the data of the mean value represent the average of four to five determinations in three independent experiments. Values in Table 3 were summarized as a mean ± standard deviation of the mean. Statistical analysis was performed using Mann–Whitney test (* *p* < 0.05). * *p* < 0.05 800 µM, 1000 µM, 2245 µM SAC vs. control, ^Δ^ *p* < 0.05, 2245 µM SAC vs. 1000 µM SAC.

## Abbreviations

AGE	Aged garlic extract
ATCC	American Type Culture Collections
casp-3	caspase-3
casp-9	caspase-9
CBS	cystathionine β-lyase
CTH	γ-cystathionase
CTL	control
DTT	D,L-dithiothreitol
NEM	N-ethylmaleimide
FC	flow cytometry
Gi	growth inhibition
Hsp90	heat shock protein 90
LDH	L-lactic dehydrogenase
MGL	methionine γ-lyase
MPST	3-mercaptopyruvate sulfurtransferase
MTT	3-(4,5-Dimethylthiazol-2-yl)-2,5-Diphenyltetrazolium Bromide
NADH	β-Nicotinamide adenine dinucleotide reduced disodium salt hydrate
PA	pyruvate
PCA	perchloric acid
PLP	pyridoxal phosphate
SAC	S-allyl- L-cysteine
SAM	S-adenosyl-L-methionine
SD	standard deviation
WB	Western blot
3MP	3-mercaptopyruvate
